# Assessment of sexual dimorphism in the humerus among a Greek Cypriot population using binary logistic regression and linear discriminant analysis

**DOI:** 10.1007/s12024-025-00984-y

**Published:** 2025-03-19

**Authors:** Erica Baer, Anna S. H. La Valley, Xenia-Paula Kyriakou

**Affiliations:** 1 Department of Psychology, Kean University, 1000 Morris Avenue, Union, NJ 07083 USA; 2 Department of Forensic Science, University of West London, Brentford, UK

**Keywords:** Sex Estimation, Forensic anthropology, Sexual dimorphism, Humerus, Metric analysis

## Abstract

**Purpose:**

Determining the sex of unknown human remains is pertinent to the reconstruction of biological profiles in forensic anthropology. The Greek Cypriot population is underrepresented in forensic anthropology literature, with only a handful of sex estimation studies having been produced thus far. The aim of this research is to provide accurate and reliable methods for estimating the sex of Greek Cypriot remains to forensically evaluate unknown human remains.

**Methods:**

This study created classification models using two statistical methods, binary logistic regression (BLR) and linear discriminant function analysis (LDA), to determine which method provided more accurate sex classification based on measurements of the humerus in a Greek Cypriot population. Additionally, cut points were calculated for use in classification. The sample consisted of 119 Greek Cypriots from the Cyprus Research Reference Collection (CRRC; 1975–2015). Four classification models were built, implementing BLR and LDA for both left- and right-side measurements. These models were analyzed using accuracy rates, receiver operating characteristic (ROC) curves, area under the curve (AUC), and Cohen’s kappa.

**Results:**

The findings revealed that all four models demonstrated good to excellent classification rates based on AUC (0.88–0.91) and accuracy rates (85.56–87.92%). Maximized summed sensitivity and specificity ratios, ranging between 1.55 and 1.76, were used to determine the optimal cut points by measurement.

**Conclusion:**

Based on these results, BLR is a better choice to evaluate sexual dimorphism of the humerus in Greek Cypriots. Further, cut points based on individual measurements can serve as useful markers for classifying humeri by sex.

## Introduction

Sex estimation is one of the most important steps in the reconstruction of a biological profile in forensic anthropology and serves as an initial assessment for establishing other biological profile factors, such as age, stature, and ancestry [1]. Forensic anthropologists assess sex in skeletal remains using both morphological analysis and osteometry. However, preference is often given to osteometric methods as they are least prone to observer bias [2–5] and allows interpretation of findings within a framework of statistical probability [6–7].

Sexual dimorphism is influenced by genes and differences in sex specific hormones between biological males and females that appear during puberty. These differences result in physiological and somatotrophic variation between males and females [8]. Sexual dimorphism is species-specific and varies between groups of the same species [9]. Human form and size in both cranial and postcranial elements are directly related to a population’s specific growth patterns [10] and are influenced by intrinsic (systemic) and extrinsic (environmental) factors. Additionally, human variation is affected by the secular changes populations undergo, suggesting that intra-population differences between males and females are also likely to develop further over time [11]. Therefore, the underlying expectation is that there is a significant amount of variation that could exist in the expression of sexual dimorphism within and between populations [9].

The Greek Cypriot population is underrepresented in forensic anthropological literature, with only a handful of sex estimation studies having been produced thus far. Kranioti et al. (2017) used linear discriminant analysis (LDA) to evaluate sexual dimorphism of the tibia in the Greek Cypriot population, finding that it is a good predictor of sex, with a classification accuracy ranging from 78 to 85% [12]. Cohen et al. studied binary logistic regression (BLR) sex estimation models for the humerus, finding classification accuracy ranged from 88 to 93% for Greek Cypriots [13]. Diaz et al. evaluated calcanei and tali of Greek Cypriots, creating models using BLR. The study found both bones to be beneficial in sex classification, with accuracy ranges of 76–80% for the calcaneus and 76–78% for the talus [14]. Relatedly, Kranioti et al. (2018) compared cranial measurements from three Mediterranean populations, inclusive of Greek Cypriots, and found that even in neighboring countries, there are distinct differences that can be used to classify skulls with relatively good accuracy [15]. This highlights the need for developing population-specific methods based on contemporary skeletal data.

Historically, the field of forensic anthropology has favored the use of LDA to evaluate sexual dimorphism in skeletal remains [16–17]. Studies have proven the usefulness of LDA in sex and ancestry classification models [18]. Even knowing the strengths and weaknesses of LDA (notably the many restrictive conditions) [19], there is a striking lack of published research comparing its effectiveness to other methods using the same population sample. More recent research has pivoted to the use of BLR to evaluate different skeletal elements [14, 20–25]. However, these studies lack comparison with LDA outcomes.

Some research does compare outcomes between models using LDA and BLR. The results of these studies vary regarding which method provides greater accuracy. In studies estimating sex from metatarsals in a South African population of African descent and mandibles in a contemporary Chinese Han population [26–27], the use of LDA showed greater accuracy. In comparison, BLR was the more accurate method in a study estimating sex from metatarsals in a South African population of European descent and a study of sterna in a Sudanese population [28–29]. On the other hand, a study estimating sex from measurements of the ear in a North Indian population found no accuracy differences based on the use of BLR or LDA [30].

The aim of this study was to quantify sexual dimorphism in the humerus of Greek Cypriots and to evaluate models for sex estimation in skeletal remains by comparing two predictive statistical methods and calculate individual measurement cut points. The objective of this study is to develop forensic sex estimation criteria for the Greek Cypriot population based on the statistical method providing the most accurate sex classification of the humerus.

## Materials and methods

The sample included 58 males and 61 females, resulting in 119 left and right humerus pairs from individuals of Greek Cypriot ancestry from the Cyprus Research Reference Collection (CRRC; 1975–2015), curated by the local Diocese of Limassol, Cyprus. Bones with visible pathological conditions or trauma were excluded from this analysis, as these factors could alter the shape, size, and other aspects of bone morphology in ways that might affect or bias the measurements obtained. Some individuals were missing measurements for their left or right humerus, resulting in 115 left (male *n* = 55; female *n* = 60) and 113 right (male *n* = 56; female *n* = 57) humeri included in the study. Eight unique measures were taken of each humerus: maximum length, maximum humeral diameter, maximum vertical head diameter, midshaft circumference, midshaft minimum diameter, midshaft maximum diameter, epicondylar breadth, and trochlear condylar breadth (Table 1). All measurements are in millimeters (mm). Permission for this study was obtained by the local Diocese authorities. The study was approved by the University of West London ethics committee (UREC/UWL/REC/PSW-01701).

**Table 1:** Measurement descriptions

Measurement	Description
Maximum vertical head diameter	The distance between the most superior and inferior points on the border of the articular surface.
Midshaft circumference	Using the half point of the Max Length Measurement, the circumference of the bone present at that location.
Midshaft maximum (perpendicular: anterior-posterior aspects)	The maximum diameter of the humeral shaft at midshaft.
Midshaft minimum (vertical: medio-lateral aspects)	The minimum diameter of the humeral shaft at midshaft.
Maximum humeral diameter	The maximum diameter of the humeral shaft.
Epicondylar breath	The distance from the most laterally protruding point on the lateral epicondyle to the corresponding projection on the medial epicondyle.
Trochlear-condylar breadth	The distance from the lateral most portion of the capitulum to the medial most portion of the trochlea.
Maximum length	The distance from the most superior point on the head of the humerus to the most inferior point on the trochlea.

Diagnostic testing generally showed a relatively normal distribution of measurements. Due to the existence of multicollinearity in the sample, two measurements, midshaft maximum diameter and maximum humeral diameter, were excluded from BLR and LDA for both left and right sides. Cook’s *d* found two individuals (one male, one female) to be outliers. Both individuals were outliers for both left- and right-side measurements. For the female outlier, most measurements were larger than 98% of the male sample. The humerus maximum length for this individual was the longest in the sample for both left and right sides and was 17 and 20 mm longer, respectively, than the next longest in the sample. For the male individual, the measurement differences were not as extreme as the female outlier. Given the sample size and the natural variation of skeletal measurements, it is normal to have one or two outliers. Both BLR and LDA are robust measures and will not be significantly impacted by their inclusion in the models.

A random selection of samples was measured twice to evaluate the reliability of the measurements taken. Interobserver reliability was assessed using intraclass correlation coefficient (ICC). The analysis revealed ICC values between 0.37 and 0.99, indicating three measurements have poor agreement between the raters (< 0.50), two have moderate agreement (0.50–0.75), two have good agreement (0.75–0.90), and the remaining five have excellent agreement (> 0.90). The variability between ICC values is high due to the small number of subjects scored by both raters (Table 2). The symmetry of left- and right-side measurements was evaluated using paired-samples *t*-tests. The measurements were also evaluated using independent-samples *t*-tests to determine sexual dimorphism individually.

**Table 2:** Interobserver error results

Measurement	Left		Right	
	ICC^a^	*n*	ICC^a^	*n*
Maximum vertical head diameter	0.95	14	0.99	12
Midshaft circumference	0.37	13	0.42	12
Midshaft maximum diameter	0.96	13	0.93	12
Midshaft minimum diameter	0.54	13	0.91	12
Trochlear-condylar breadth	0.49	13	0.60	12
Maximum length	0.87	12	0.87	12

^a^Intraclass correlation coefficient (ICC)

Models using both LDA and BLR were created for both left- and right-side measurements using k-fold cross validation. K-fold cross validation is a common resampling method used to estimate how well a model performs. In this study, 10 subsamples were used to train the model. A hold-out group of 25% of the sample was then used to test the accuracy of the models created via cross validation. This iterative process using k-fold cross validation allows for a more reliable estimate of model performance.

The models were evaluated using accuracy rates, receiver operating characteristic (ROC) curves, area under the ROC curve (AUC), and Cohen’s kappa. Accuracy rates can be defined as the number of correctly predicted individuals out of the total number of cases. A ROC curve is a visual representation of how well the model can classify the data based on the true positive rate and the false positive rate. Relatedly, AUC is defined as the area underneath the ROC curve and provides a measure of performance for the model. An AUC of 1.0 shows that the model will correctly classify all individuals in the sample. Cohen’s kappa provides a measure of agreement between the model predictions and actual results. Similar to AUC, a score of 1.0 shows perfect agreement between the model predictions and actual results.

Finally, the data were evaluated to develop cut points for the classification of remains based on each measurement of the humerus. A cut point is a specific value used for categorization. Each set of measurements was evaluated separately to determine the optimal cut point to distinguish between male and female remains. For this analysis, cut points were selected by maximizing the sensitivity and specificity for each measurement. Sensitivity is defined as the true positive rate. For this study, this is the proportion of cases that are accurately classified as female by the model. Specificity is defined as the true negative rate. For this study, this is the proportion of cases that are accurately classified as male. By maximizing the sum of the sensitivity and specificity, we can ensure that the greatest number of cases are correctly classified. All analyses were conducted with R Studio 4.4.1.

## Results

The data were evaluated using paired-samples *t*-tests to evaluate the bilateral symmetry of the left and right measurements of the humerus. The tests found significant differences between left and right humeri in four of the eight measurements (Table 3). The eight measurements in this study were also evaluated individually for sexual dimorphism using independent-samples *t*-tests. The mean for each measurement was significantly larger for male individuals than female individuals (Table 4). Descriptive analysis of the eight measurements can be seen in Table 5.

**Table 3:** Symmetry of left- and right-side measurements using paired-samples *t*-test

Measurement	Mean difference (95% CI)	t
Maximum vertical head diameter	0.12 (-0.32, 0.57)	0.55
Midshaft circumference	0.71 (0.32, 1.10)	3.58***
Midshaft maximum diameter	0.12 (-0.03, 0.27)	1.58
Midshaft minimum diameter	0.25 (0.02, 0.47)	2.16*
Maximum humeral diameter	0.12 (-0.04, 0.29)	1.54
Epicondylar breadth	0.76 (0.19, 1.34)	2.64**
Trochlear-condylar breadth	-0.36 (-0.95, 0.23)	-1.21
Maximum length	2.15 (0.91, 3.40)	3.42**

* *p* <.05, ** *p* <.01, *** *p* <.001

**Table 4:** Sexual dimorphism in individual measurements using independent-samples *t*-test

Measurement	Left		Right	
	t	*p*	t	*p*
Maximum vertical head diameter	7.14	< 0.001***	5.93	< 0.001***
Midshaft circumference	6.94	< 0.001***	6.57	< 0.001***
Midshaft maximum diameter	6.28	< 0.001***	6.15	< 0.001***
Midshaft minimum diameter	7.95	< 0.001***	7.03	< 0.001***
Maximum humeral diameter	6.69	< 0.001***	6.85	< 0.001***
Epicondylar breadth	8.55	< 0.001***	8.88	< 0.001***
Trochlear-condylar breadth	7.95	< 0.001***	7.41	< 0.001***
Maximum length	8.72	< 0.001***	8.30	< 0.001***

*** *p* <.001

**Table 5:** Right- and left-side humerus measurements in males and females

Measurement (mm)	Males		Females	
	Left	Right	Left	Right
	Mean (SD)	Mean (SD)	Mean (SD)	Mean (SD)
Maximum vertical head diameter	44.73 (3.52)	44.74 (3.73)	39.96 (3.62)	40.53 (3.82)
Midshaft circumference	64.51 (5.81)	65.32 (6.01)	56.98 (5.83)	58.16 (5.93)
Midshaft maximum diameter	21.64 (1.72)	21.77 (1.81)	19.56 (1.90)	19.78 (1.81)
Midshaft minimum diameter	19.39 (1.88)	19.60 (1.94)	16.57 (1.84)	17.09 (1.92)
Maximum humeral diameter	21.98 (1.69)	22.24 (1.75)	19.80 (1.88)	20.03 (1.82)
Epicondylar breadth	61.82 (3.87)	62.00 (3.99)	54.31 (5.09)	55.89 (3.41)
Trochlear-condylar breadth	45.31 (3.30)	44.34 (3.12)	39.40 (4.44)	39.84 (3.12)
Maximum length	313.17 (14.39)	315.66 (14.56)	285.70 (19.53)	288.33 (19.24)

**Fig. 1:**
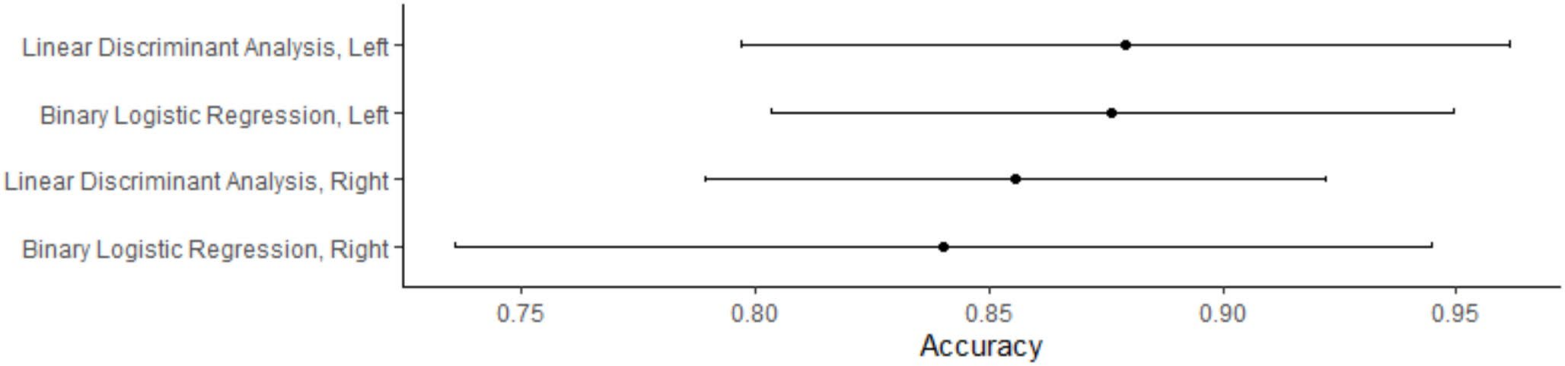
The accuracy rates achieved by each model are shown with its 95% confidence interval. A wider confidence interval indicates a greater amount of uncertainty in model accuracy

For left-side measurements, BLR found maximum length to be the only significant predictor (*p =*.041). For right-side measurements, BLR found two significant predictors, maximum length (*p =*.008) and epicondylar breadth (*p* =.046). The remaining predictor variables for both the left and right sides did not significantly impact the model. However, removing nonsignificant measurements reduced the accuracy of the models. Therefore, all remaining measurements were included in the final BLR and LDA models. All four models performed well, showing good to excellent classification accuracy based on AUC, accuracy rate, and Cohen’s kappa (Table 6). The models using left-side measurements appear to have slightly outperformed the models using right-side measurements when viewing a comparison of accuracy rates with 95% confidence intervals (Fig. 1).

**Table 6:** Comparison of performance of LDA and BLR models using AUC, accuracy rates, and Cohen’s kappa

Model	AUC	Accuracy	Kappa
**Binary Logistic Regression**,** Left**	0.89	0.8763	0.7519
**Linear Discriminant Analysis**,** Left**	0.91	0.8792	0.7580
**Binary Logistic Regression**,** Right**	0.88	0.8403	0.6800
**Linear Discriminant Analysis**,** Right**	0.89	0.8556	0.7076

**Fig. 2:**
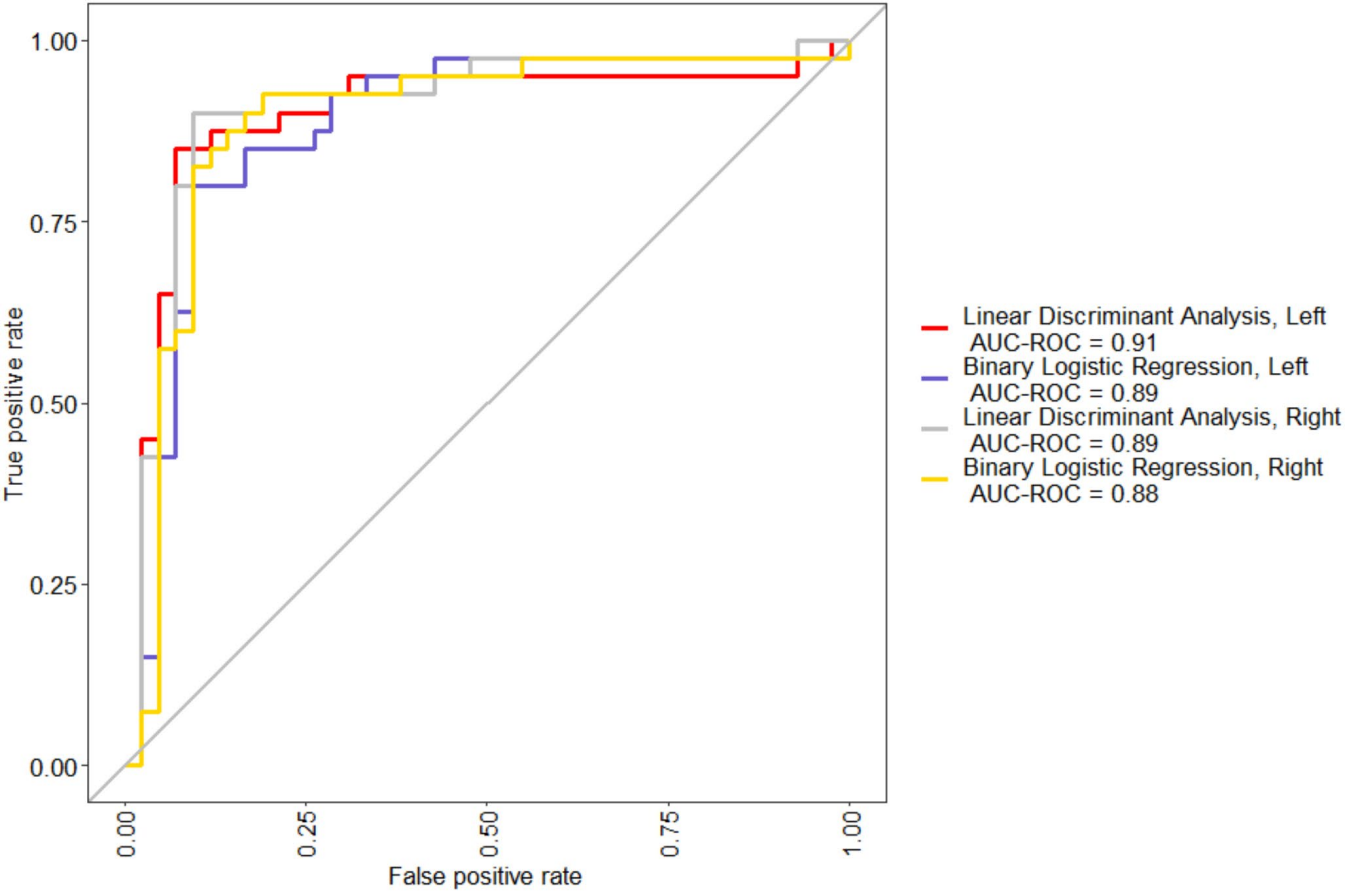
Receiver operating characteristic curves for the four models. The lines indicate the false positive rate (individuals inaccurately predicted to be female; x-axis) and true positive rate (individuals accurately predicted to be female; y-axis) for each model. Models that have curves closer to the top-left corner of the graph indicate greater accuracy

Using the ROC curves shown in Fig. 2, a visual analysis of the models shows that all four models performed similarly. The left-side BLR model used in this study slightly outperformed the right-side model, suggesting the left-side model using BLR to be the best option for developing classification criteria for this sample. The classification equation developed for the BLR using left-side measurements for this data is

$$\begin{array}{l}\\\:Log\left(\widehat{y}\right)=-35.876-0.103\left(LMVH\right)+0.036\left(LMC\right)\\+0.048\left(LMM\right)+0.315\left(LEB\right)+00054\left(LTB\right)+0.055\left(ML\right)\end{array}$$

In addition to using ROC curves to compare the classification models using BLR and LDA, ROC curves can also assist with the determination of optimal cut points. Using left-side measurements, which were found to provide greater accuracy, cut points were selected by maximizing the sensitivity and specificity for each measurement. In this study, humeri from males have larger average measurements than females, meaning that measurements larger than the cut point were classified as male, while measurements below the cut point were classified as female. Sensitivity using this method ranged between 0.6250 and 0.8929 and specificity ranged between 0.8302 and 0.9245. The epicondylar breadth and trochlear-condylar breadth emerged as the measurements with the highest maximized sensitivity and specificity. The full list of cut points and associated sensitivity and specificity ratios can be found in Table 7.

**Table 7:** Optimal cut points based on maximization of sensitivity and specificity

Measurement	Cut Point (mm)	Sensitivity	Specificity
Maximum vertical head diameter	42.1	0.8571	0.8302
Midshaft circumference	59.0	0.7321	0.8679
Midshaft maximum diameter	19.7	0.6250	0.9245
Midshaft minimum diameter	17.4	0.8214	0.8679
Maximum humeral diameter	20.2	0.6964	0.8679
Epicondylar breadth	57.6	0.8571	0.8868
Trochlear-condylar breadth	42.3	0.8929	0.8679
Maximum length	300.0	0.8571	0.8302

## Discussion

Accurate classification of unknown skeletal remains into sex categories is fundamentally important in any forensic setting. The results of this study found that there is a good degree of skeletal sexual dimorphism between Greek Cypriot males and females. All four models in this study provided good to excellent classification rates based on AUC (0.88– 0.91) and accuracy rates (85.56–87.92%). Maximum length emerged as a significant predictor of sex in all models, suggesting its importance in accurate sex classification. The current study suggests both BLR and LDA are potentially useful tools in evaluating sexual dimorphism. The models developed in this study are specific to the Greek Cypriot population. However, the process used in this study can be considered in the evaluation of other Mediterranean or European populations.

Given the methodological differences between BLR and LDA and the results above, BLR for left-side measurements provided the best evaluation for the current sample. In general, LDA is a more powerful and more efficient classification tool in comparison to BLR when assumptions are met, and the outcome variable is binary. However, when sample sizes are small, the robustness of a LDA cannot be assured [31]. BLR provides a more versatile analysis with fewer limitations when assumptions are not met.

Further, there is a growing consideration that BLR may be a more successful classification method due to the differing structures of the methods [32]. In general, BLR determines the probability of a chosen outcome event occurring by estimating the parameters based on the predictor variables. For this study, BLR analysis was used to estimate the outcome variable (sex) given the assumption that each set of remains was equally likely to be male or female. For BLR, the necessary assumptions are (1) the measurements must be independent of each other, (2) there is a lack of multicollinearity, (3) there are no influential points, and (4) there is a linear relationship between the predictor variables and the logit of the response variable. In LDA, group differences are modeled through dimension reduction, necessitating a different set of assumptions from BLR. For LDA, the assumptions are that (1) the measurements must be independent of each other, (2) be normally distributed, and (3) have similar variances. Typically, researchers find BLR to be a more robust and more flexible method due to the more stringent requirements of LDA [32–33].

These results provide a similar outcome to previous research evaluating the use of BLR and LDA in sex estimation. Research in the field of forensic anthropology has used LDA more often than BLR in the past, however recent research shows BLR to perform as well as, or better than, LDA [17, 27–30, 33]. In a comparison of BLR and LDA, research found BLR to classify cases more accurately while relying on fewer assumptions [33]. Previous research has also suggested while either BLR or LDA might lead to more accurate results [26–29], using both methods in tandem can provide useful classification models that can benefit researchers and practitioners when classifying remains by sex.

In addition to the use of predictive statistical measures, cut points can also be beneficial to the classification of remains. Several methods have been developed to determine optimal cut points, based on the data being evaluated and aims of the research [34–35]. Given these findings, the maximization of the sum of the sensitivity and specificity provide the optimal cut point for classifying remains by sex. The combination of predictive methods, along with optimal cut points, provides researchers and practitioners significant guidance in this endeavor.

Classification rates varied slightly between BLR and LDA in this study. The BLR and LDA models using left-side measurements were both more accurate in predicting sex than their right-side counterparts. In general, research suggests that neither BLR nor LDA consistently outperforms the other [36]. There are situations where BLR is the appropriate choice, such as when predictor variables are not normally distributed or when the number of outcome categories is small. Based on these differences, and the findings of our study, it is recommended to use BLR with left-side measurements when classifying individuals of Greek Cypriot ancestry. While LDA also provided a useful model based on left-side measurements, the benefits of using BLR noted above cannot be overlooked. In addition to classification by BLR, the cut points calculated in this study can act as an initial starting point for classification. Female humeri produced smaller measurements on average, than humeri from male individuals. These differences were statistically significant for all measurements, thus determining that the humerus is characterized by a degree of sexual dimorphism. The cut points for the individual measurements can be used as a quick screening tool to evaluate the likelihood of classifying a humerus as coming from a male or female individual.

This study also adds to the small, but growing, body of research in forensic anthropology focused on the Greek Cypriot population. Recent studies including Greek Cypriots have focused on ancestry estimation, using sex estimation to assist in these analyses. Evaluation of the metric variation in the tibia and cranium found significant sex differences using different statistical methods than the ones employed in the current study, including independent-samples *t-*test, Mann-Whitney U, Wilcoxon W, and LDA [14, 15, 38]. Research focused on sexual dimorphism of the humerus, talus, and calcaneus in Greek Cypriots showed BLR to be a useful metric in classification by sex [13–14].

Some limitations regarding this study should be noted. This research focused on a small sample from a single, homogeneous population. This was an important feature of the current study; however, it does limit the potential generalizability for these findings beyond the current population. Considering the sample’s extensive temporal range, the potential for secular change must be acknowledged. Secular changes occur over decades or generations and are primarily influenced by environmental factors such as diet, climate, and lifestyle [37]. Secular change could therefore influence the accuracy and reliability of metric sex estimation methods in forensic anthropology when not evaluated. However, in this study the temporal window may also be considered short (< 50 years), given that it is less than two biological generational cycles [38]. Additionally, the two individuals considered influential points could be reviewed to further evaluate population variation. Outliers are likely to occur through natural variations in the population. Given that only two outliers emerged in this dataset, it is important to retain them in the dataset to develop accurate models for the Greek Cypriot population. Finally, including a greater number of samples in the interobserver reliability analysis would provide more certainty in the accuracy of the measurements.

## Conclusion

The findings of this study have significant implications for forensic cases, emphasizing the need for updated metric sex estimation methods tailored to specific populations. Implementing these population-specific methods can enhance the efficiency of human identification processes and lead to more accurate sex determinations for unknown skeletal remains. As noted, metric methods are preferred over morphological methods for sex estimation. Long bones are far less affected by the age-related changes that occur in the cranium and pelvis and their increased cortical robusticity and overall structural geometry make long bones more durable against taphonomic alterations. Thus, long bones are more likely to be preserved for forensic analysis.

Sex estimation studies establish a methodological foundation for human identification, supporting medicolegal death investigations on the island. Future research on sexual dimorphism of the humerus could expand in several promising directions. One avenue is to apply the current methodology to larger samples. This could potentially increase the generalizability of findings and increase opportunities to apply these methods to other populations. The integration of machine learning could enhance sex determination methods for the humerus by detecting complex patterns beyond traditional statistics and thus could enhance model accuracy. Lastly, it is recommended that future research should explore BLR application to other bones or bone combinations from this population sample, aiding sex classification when humeral length is unknown or unreliable.

In conclusion, both BLR and LDA showed high rates of accuracy in classifying the humeri in this sample by sex. The results indicate that both methods can be useful tools in sex classification of the humerus. This finding is in agreement with other published studies. Based on the size of the sample and the versatility of BLR, the use of BLR is recommended when classifying humeri from individuals of Greek Cypriot ancestry. Given the data in this sample, left-side measurements provide slightly more accurate classification. The BLR equation and individual cut points derived in this study can be used to classify remains of individuals of Greek Cypriot ancestry by sex.

### Key points

1. This study evaluated the effectiveness of statistical classification models in assessing sexual dimorphism based on humeral measurements.
2. Humeral length emerged as the most influential predictor of sex from among the humeral measurements analyzed.
3. Models using BLR for left-side measurements provide the most accurate sex classification results based on humerus measurements.
4. Cut points provide sex classification metrics for measurements of the humerus.

## Abbreviations

AUC: Area under the curve
BLR: Binary logistic regression
CRRC: Cyprus Research Reference Collection
ICC: Intraclass correlation coefficient
LDA: Linear discriminant analysis
NIST: National Institute of Standards and Technology
ROC: Receiver operating characteristic
